# Molecular Mechanisms of Adaptation to Hypoxia

**DOI:** 10.3390/ijms24054563

**Published:** 2023-02-26

**Authors:** Elena Rybnikova, Ludmila Lukyanova

**Affiliations:** 1 I.P. Pavlov Institute of Physiology RAS, St. Petersburg 199034, Russia; 2 Institute of General Pathology and Pathophysiology, Moscow 125315, Russia

Oxygen is one of the most important elements, ensuring the vital activity of the body. It is a key component of aerobic energy metabolism and supplies cells with macroergic ATP molecules. Therefore, a lack of oxygen, hypoxia, can have serious negative consequences and is considered a pathogenic factor in many diseases. On the other hand, in evolution, living beings experienced hypoxia even at the stage of unicellular organisms and, as a result, have formed effective mechanisms of adaptation to it that are among the most phylogenetically ancient and, therefore, reliable. These mechanisms are triggered when the oxygen level decreases, and they provide multilevel physiological adjustments that allow the maintenance of vital activity in hypoxic conditions. Thus, on the one hand hypoxia is a negative factor that causes energy deficiency and oxidative stress, and on the other it is a factor that triggers adaptive reactions. The main question is how to draw the line between positive and damaging hypoxias. This is possible only with a detailed understanding of the events occurring at the molecular level during the development either of hypoxic damage or adaptive reactions. This Special Issue is aimed at integrating a number of notable recent achievements in this area of research to stimulate further progress towards understanding the boundary between hypoxia as a pathological and hypoxia as an adaptative factor, as well as identifying the molecular mechanisms mediating the mutual transformation of the proadaptive effects to the pathological ones.

The most important questions in such studies concern the development of reliable physiological models to examine hypoxia-related cellular processes. Analyzing the relationship between aldosterone, the HIF-1 pathway and epithelial sodium channels in the kidney cortical collecting cells, Keppner and colleagues [1] have experimentally proved that these cells can serve as such a model. In particular, using the mouse cortical collecting duct cell line mCCDcl1, which represents polarized epithelial cells, exhibiting both aldosterone- and corticosterone dependent sodium transport, they have demonstrated that these cells responded to hypoxia by up-regulating the HIF-1 target genes, with HIF-1α, rather than HIF-2α, mediating this hypoxic response at the target gene level. In addition, these authors have revealed that mCCDcl1 cells can also be used to search for protective factors against kidney damage caused by hypoxia. Their findings have suggested that the newly identified protective transcription factor, namely estrogen-related receptor alpha (Esrra), can act as a cofactor of HIF-1, thereby contributing to the implementation of adaptive reactions triggered by HIF-1, at least in the mCCD cells. However, a question still remains as to whether Esrra is a universal cofactor of HIF-1, and whether a similar phenomenon can be observed in other cell lines and tissues, both in vitro and in vivo.

In the paper by Nascimento-Filho and co-authors [2], a new method to culture cells at different oxygen levels using deoxidizing absorbers is presented. A feature of the method is the ability to not only maintain in cell cultures a low level of oxygen close to the physiological norm (physioxic level), but also to gradually reduce it even further (hypoxia modeling). The results demonstrated that the cultivation of cancer cells under hypoxia conditions led to the activation of the epithelial–mesenchymal transition, increasing the expression of HIF-1a. An exacerbation of the oncogenic pathway of PI3K was also revealed in comparison with tumor cells growing at atmospheric oxygen levels. At the same time, there was an increase in oxidative stress and superoxide levels, as well as an increase in cell cycle arrest. The cultivation of cancer cells under hypoxia conditions led to the accumulation of cancer stem cells, depending on time, thus, the hypoxic environment enhanced the oncogenic effect. The presented method of oxygenation during cell culture is cost-effective and can be easily implemented.

The study of Suzuki and co-authors [3] is also based on the successful experimental application of a three-dimensional (3D) in vitro model, associated with spontaneous O_2_ gradients. The conventional 2D and this original 3D model were used to create pathological hypoxic conditions in order to study the effect of hypoxia on transforming growth factor-β2 (TGFß2)-induced epithelial mesenchymal transition (EMT) of human retinal pigment epithelium (HRPE) cells, a process that makes a significant contribution to the pathogenesis of proliferative retinal diseases. The results obtained by Suzuki and colleagues indicate that TGF-β2-induced EMT of both 2D and 3D cultured HRPE cells was significantly modified by simulated pathological hypoxia. TGF-β2 and hypoxia synergistically but diversely induced EMT of the HRPE cells, judging by the fluctuations in HIF-1α and target genes mRNA expression, as well as mitochondrial metabolism. The effects also differed between the 2D and 3D cultures, indicating some variability during the spatial spreading of the cells. The authors hypothesized that this may be caused by possible upstream regulators requiring the generation of the 3D spheroids, but this question still needs further study.

To date, the interest in the application of hypobaric hypoxia (HH), both natural or simulated in a pressure chamber, to study the processes of hypoxic adaptation and maladaptation has not faded. Thus, Arriaza and colleagues [4] provided a comprehensive review of the accumulated literature relevant to the current classification of the HH modes, as well as of physiologic and pathologic responses of the organism to HH. It is important that the authors draw the reader’s attention to the fact that HH cannot be equated with other types of hypoxia with a similar level of oxygen deficiency, including normobaric. Low pressure is also a contributing factor to the changes in the physiological processes enhancing the impact of the hypoxic factor itself. However, the focus of this review is hypoxic pulmonary vasoconstriction (HPV), representing one of the physiological compensation mechanisms in hypoxia, aimed at redistributing the blood to more ventilated areas of the lung. It is well-known that HPV overresponse can underline the pathogenesis of high-altitude pulmonary hypertension (HAPH). Analyzing the extensive literature on the mechanisms of HPV, Arriaza and co-authors indicated the role of elevated Zn^2+^ levels and oxidative stress in the development of HPV in different models of hypobaric hypoxia. This conclusion suggests possible therapeutic targets for the elimination of excessive HPV as a mechanism inducing the development of HAPH.

An important factor that largely determines the level of translation of cell proteins in response to hypoxia are post-transcriptional processes, in particular the regulation of functional dynamics of RNA. Comparing the effects of acute and chronic hypoxia in monocytic THP-1 cell culture, Bauer and colleagues [5] have described in detail the profiles of differential gene expression. At the level of de novo mRNA synthesis, only minor changes, pointing to the attenuation of hypoxia-induced transcription under chronic conditions, have been revealed. The data by Bauer and co-authors also indicated that most of the mRNAs are destabilized under hypoxia and, again, only slight changes between the effects of acute and chronic hypoxic conditions have been detected. Based on these findings, the authors have concluded that general adaptations during the course of hypoxia appear to be determined largely at a transcriptional level, but post-transcriptional mRNA destabilization also plays an important role, affording a reduction in the translation of mitochondrial proteins and, thus, modifying the mitochondria functioning under extended hypoxia.

The study by Baranova and co-authors [6] is focused on an oxygen-saving reaction such as a diving reflex, representing a complex adaptive physiological response, including changes in the cardiorespiratory function, erythropoiesis, peripheral vasoconstriction and the redistribution of the blood circulation. The molecular mechanisms of the diving reflex as an evolutionarily gained form of systemic adaptation to hypoxia have so far been understudied. The results reported by Baranova and colleagues in this Special Issue describe the polymorphisms of the α 1A adrenoceptor (ADRA1A) gene in humans, correlating with the most prominent adaptive reaction to diving hypoxia or high risks to develop lung hypertension.

It is well-known that prenatal hypoxia is one of the most common pathologies of embryonic development, which has long-term negative health consequences throughout the subsequent life, including premature aging. Based on the analysis of the accumulated evidence, the review by Sutovska and co-authors [7] aims to emphasize the role of prenatal hypoxia in the formation of hypertension in adulthood. The authors convincingly conclude that even short-term prenatal hypoxia significantly affects the mechanisms of regulation of the cardiovascular system, programming the development of hypertension in adulthood. This effect of the prenatal hypoxia depends on the critical period of pregnancy, sex and, probably, the time of day.

Recently it has been hypothesized that ferroptosis might be a possible key injury mechanism in neonatal hypoxic-ischemic brain injury. This question is extensively reviewed for our Special Issue by Peeples and colleagues [8]. Their review suggests that the attenuation of changes associated with ferroptosis after injury may be neuroprotective. In this respect, Peeples and colleagues discuss current unresolved relevant issues that need to be studied.

One of the most negative roles of hypoxic signaling, in particular related to HIF-1, is its persistent hyperactivation in tumors, leading to increased malignancy, vascularization and resistance of the cancer cells to anticancer therapy. One of the most malignant types is an androgen-dependent triple-negative breast cancer, in which hypoxic signaling provides resistance to therapy aimed at androgen or androgen receptor signaling. Analyzing the accumulated data, Jinna and colleagues [9] suggested that dual targeting of the hypoxia signaling pathway and androgen receptors may provide a promising strategy to decrease the resistance to therapy in patients with this disease.

Along with the reports on the facts testifying to the pathogenic role of HIF-1 and its down-stream hypoxic signaling, convincing data on its positive effects continue to accumulate. In particular, Cirillo and co-authors [10] have found a decrease in the expression of HIF-1α and its target genes in sarcopenia. The findings allow us to expect that pharmacological reactivation of HIF-1α could prevent and treat sarcopenia.

Along with the pathological consequences of hypoxia, it is important to pay special attention to the protective effects of its moderate forms. Studies of such effects and the protective hypoxia regimes are important not only for fundamental research, but also with regard to the practical potential of using such regimes to improve health, as well as to increase body resistance to pathogenic factors. In this regard, interesting data have been published in our Special Issue by Luo and colleagues [11], demonstrating that the daily training of obese mice using moderate hypoxia (10% of O2) alleviated the pathogenic effects of a high fat diet. In particular, hypoxic training prevented bodyweight gain and development of insulin resistance, reversed the enlargement of white and brown adipocytes and protected liver function. A high fat diet downregulated the expression of genes required for lipolysis and thermogenesis, while these molecules were upregulated by hypoxia. The authors revealed that hypoxia, being a stress factor, significantly increased the serum levels of epinephrine, and then proved experimentally that the up-regulation of epinephrine represents a key factor mediating the observed therapeutic effect of hypoxia via the catecholamine-PKA-AMPK pathway. The findings reveal for the first time the therapeutic potential of hypoxic training in the treatment of obesity, nonalcoholic fatty liver disease and other metabolic disorders.

In conclusion, the articles published in the Special Issue “Molecular Mechanisms of Adaptation to Hypoxia” make a significant contribution to the current knowledge on the mechanisms of hypoxia and our adaptation to it. However, there are still many topical issues left outside the scope of this Special Issue, which we hope to cover very soon in the next Special Issue: “Molecular Mechanisms of Adaptation to Hypoxia 2.0”.

## Data Availability

Not applicable.
